# Pseudohypoaldosteronism Type 1: The Presentation and Management of a Neonate With a Novel Mutation of the SCNN1B Gene Found in Two Hispanic Siblings

**DOI:** 10.7759/cureus.23918

**Published:** 2022-04-07

**Authors:** Charles P Pugh

**Affiliations:** 1 Pediatrics/Neonatology, University of Arkansas for Medical Sciences, Little Rock, USA

**Keywords:** pha type 1, pha gene variant, neonatal salt wasting, scnn1b gene, pseudohypoaldosteronism type 1

## Abstract

Pseudohypoaldosteronism type 1 (PHA1) may manifest in the neonatal period as a life-threatening salt-wasting syndrome providing challenges in recognition and treatment. This case describes a newborn who developed severe dehydration and electrolyte imbalances and subsequently was found to have a novel SCNN1B gene variant resulting in autosomal recessive systemic PHA1.

## Introduction

Pseudohypoaldosteronism type 1 (PHA1) is characterized by features including poor renal excretion of potassium, sodium salt wasting, and severe volume depletion resulting in hyponatremia, hyperkalemia, and metabolic acidosis with high serum aldosterone concentrations. This rare disorder consists of two distinct forms of mineralocorticoid resistance either renal (autosomal dominant) or systemic (autosomal recessive) which are distinguished on clinical and genetic levels [1]. The autosomal dominant (ad-PHA1) form is thought to be a milder disease that remits with age and is the result of a heterozygous mutation of the mineralocorticoid receptor gene NR3C2 (nuclear receptor subfamily three group C member 2) [2]. On the other hand, the autosomal recessive (ar-PHA1) form is characterized by multi-organ involvement and more severe neonatal onset salt wasting resulting from reduced function of epithelial sodium channels (ENaC) secondary to mutations in any one of three genes (SCNN1A, SCNN1B, and SCNN1G) which are sodium channel epithelial genes [1]. As such, infants with the systemic form exhibit multi-organ salt loss and often have pulmonary and respiratory complications which can be devastating and life-threatening. Since the first description of this disease in 1958, several reported case studies have broadened the knowledge of this inherited disorder, but clinical presentation and management continue to present challenges [3]. In this case, we report the presentation, clinical course, diagnosis, and challenges of treatment of a patient with systemic PHA1. Additionally, we report a previously unreported novel genetic variant of systemic PHA1 found in both the patient and sibling.

## Case presentation

The patient was a 5-day-old Hispanic male who was admitted from an outside emergency room with hyperkalemia and decreased oral intake. He was a product of a 41-week, singleton gestation with non-consanguineous parents born 3.9 kg at birth to a 28-year-old Hispanic mother. He had an uncomplicated delivery and was discharged home at 2 days of life with plans to follow up in a genetics clinic due to an older sibling, a 19-month-old Hispanic male, having a previous diagnosis of PHA1 (autosomal recessive). Laboratory results before initial discharge on the day of life 2 were normal (Table 1). The patient was discharged after taking 2 ounces of regular expressed breast milk every 2 hours until day of life 5 when parents noted the infant to be more listless and had decreased intake by mouth for over 4-6 hours before being taken to an outside emergency room. On arrival at the emergency room, the patient’s weight was 3.185 kg, down 18% from birth weight. Initial laboratory results on day of life 5 were concerning for hyperkalemia (Table 1). Electrocardiogram was normal sinus rhythm with intermittent aberrant ventricular conduction and no evidence of peaked T waves. Patient was given normal saline bolus 20 ml/kg and started on D10 water at 100 ml/kg/day and transferred to higher level of care newborn intensive care unit (NICU) with 2 mg IV hydrocortisone given en route. On arrival at NICU, the patient was given multiple doses of albuterol, calcium, bicarbonate, insulin/glucose, and increased in total fluid goal to two times maintenance of IV fluids with normal saline as the patient had severe dehydration. On physical exam, the patient had an erythematous rash on the face and chest but no signs of abnormal or ambiguous genitalia. The patient was initially admitted on fludrocortisone for salt wasting and concern for adrenal insufficiency but this was weaned over the next few days as an inpatient. Initial laboratory results were consistent with salt-wasting (Table 2). The newborn screen for congenital adrenal hyperplasia was negative. Renal ultrasound was normal with normal color and spectral doppler evaluation. Genetic testing was performed involving analysis of the coding region of regions of SCNN1A, SCNN1B, SCNN1G, and NR3C2. The molecular genetics targeted testing by laboratory prevention genetics using Sanger sequencing revealed a mutation of the SCNN1B gene, exon 4 homozygous variant for c.682del, which is predicted to result in a frameshift and premature protein termination (p.Ala228Hisfs*8). To current knowledge, this specific variant has not been reported and is absent from large population databases [4]. This variant is expected to be pathogenic for PHA1 (autosomal recessive) based on clinical phenotype and genetic locus. The patient’s brother also had a previous diagnosis of PHA1 by genetic sequencing with the same mutation of gene transcript SCNN1B homozygous variant c.682del frameshift chr16:23366716.

**Table 1:** Electrolytes at Day of Life 2 and Day of Life 5 in an Infant with Concern for Pseudohypoaldosteronism Type 1

	Day of Life 2	Day of Life 5
Sodium	141 mmol/L	133 mmol/L
Potassium	5.4 mmol/L	10.4 mmol/L
Chloride	107 mmol/L	101 mmol/L
Bicarbonate	20 mmol/L	15 mmol/L
Blood Urea Nitrogen	18 mg/dL	21 mg/dL
Creatinine	0.5 mg/dL	0.7 mg/dL
Glucose	72 mg/dL	97 mg/dL

**Table 2:** Infant’s Initial Workup for Concerns for Pseudohypoaldosteronism Type 1

	Infant’s Workup	Normal Value Range
Aldosterone	1086 ng/dL	(5-175 ng/dL)
Cortisol	>61.6 µg/dL	(0.6-19.8 µg/dL)
17-hydroxyprogesterone	26 ng/dL	(<78 ng/dL)
Urine osmolality	583 mOsm/kg	(800-1400 mOsm/kg)
Urine sodium	187 mmol/L	(3-35 mmol/L)

During the hospitalization, the patient subsequently needed an increase in sodium supplements and kayexalate decanting of formula to maintain electrolytes within normal limits. Sodium supplementation was added in small increasing fractions to see if the patient could develop a tolerance for taste of the formula. The patient could not reach full volume oral feeds, hence, gastrostomy tube feeds and sodium supplementation were introduced. This allowed for needed volume of feeds and supplementation to achieve normalized electrolytes prior to discharge. Since discharge, the patient has continued to have eczematous skin rash and electrolytes have been monitored with supplementation adjusted as needed. Since discharge, the patient also has been hospitalized for viral bronchiolitis positive for adenovirus and pneumonia infections with concern for recurrent respiratory problems early in life. At the 6-month clinic visit, patient’s weight, height, and BMI were 7.15 kg 30% (Z= −0.52), 65.5 cm 37% (Z= −0.32), 16.67 kg/m² 33% (Z= −0.45), respectively based on World Health Organization (WHO) (Boys, 0-2 years). Supplementation included kayexalate 9 g daily, added to 24-hour feed volume and feed supernatant subsequently decanted, sodium chloride 84 meq/day, and sodium bicarbonate 3 meq/day.

## Discussion

PHA1 is a disorder that presents in neonates with severe volume depletion and hyponatremia, hyperkalemia, and metabolic acidosis with high levels of aldosterone which poses a significant threat to life in these patients. The initial presentation of PHA1 is similar to the presentation of congenital adrenal hyperplasia (CAH) which is the more common salt-wasting syndrome, and often patients will initially be treated with mineralocorticoid hormones without improvement [5]. Due to the severity of outcomes and the need for rapid electrolyte correction that remains unresponsive to mineralocorticoid hormones, expanding our knowledge and recognition of PHA1 is essential.

PHA1 is characterized by two distinct forms of mineralocorticoid resistance either renal (autosomal dominant) or systemic (autosomal recessive) [1]. The autosomal dominant form is thought to be a milder disease that is characterized by salt loss exclusively through the kidneys. The cause of this autosomal dominant form is from a heterozygous mutation of the mineralocorticoid receptor gene NR3C2 [1,2]. In most patients with autosomal dominant PHA1, sodium supplementation can be discontinued by age 1-3 in children which is thought to be secondary to salt conservation [1].

In contrast, autosomal recessive systemic PHA1 form is a multi-organ life-threatening disorder with severe salt-wasting and high concentrations of sodium in sweat, stool, and saliva [3]. With multi-organ involvement, these patients develop a cystic fibrosis-like pulmonary dysfunction and are prone to recurrent respiratory problems [6]. Patients with systemic PHA1 are also prone to develop an atopic dermatitis rash. This is thought to be secondary to inflammation in the eccrine structures from increased salt loss through the skin [7]. This case report follows other reported cases phenotypically with similar atopic features and additionally shows the risk of recurrent infections in this population as the index patient has had multiple hospital admissions for respiratory illnesses.

The autosomal recessive PHA1 form is caused by a mutation in one of three genes (SCNN1A, SCNN1B, and SCNN1G) encoding epithelial sodium channels (ENaC) [8]. Genetic defects and mutations located throughout the SCNN1B have been described including missense, splicing, nonsense, deletions, and insertions as seen in large population databases [4]. The variant in the index case is a previously unreported novel mutation involving the SCNN1B gene, exon 4 sequencing revealing a homozygous variant for c.682del. This is predicted to result in a frameshift and premature protein termination (p.Ala228Hisfs*8). The patient’s brother also had genetic sequencing revealing a mutation of gene transcript SCNN1B variant c.682del frameshift chr16:23366716. Both patients have followed a similar clinical course demonstrating similar phenotypes and this variant is considered pathogenic for systemic PHA1.

Additionally, treatment and medical management of systemic PHA1 continue to pose a serious difficulty. These patients will often require lifelong sodium supplementation with the goal to maintain normal range plasma sodium and potassium within range which is often difficult to achieve. In PHA1, the mechanism of dysfunction of the distal collecting kidney tubules is secondary to genetic mutations that result in the inability to reabsorb sodium which is necessary for potassium excretion [9]. Thus, baseline treatment is aggressive sodium supplementation usually much higher than normal physiologic needs and potassium excretion by formula depleted of potassium or use of kayexalate to further excrete potassium. Patients will also need further sodium bicarbonate supplementation for metabolic acidosis.

As such, a particular challenge and pitfall in the management of neonates with PHA1 as seen in this case report is the need for such high sodium supplementation and volume of feeds. This case demonstrates how even a short period of decreased oral intake can lead to serious electrolyte abnormalities. As a result, other options for consistent management must be explored. During the hospital stay, this patient was not able to take goal volume or full oral sodium supplementation needed to maintain normal electrolytes. A gastrostomy tube was placed and offers a solution to this step in management. This allowed for consistent titration of both volume of feeds and electrolyte supplementation as an outpatient lowering the risk of the infant returning severely dehydrated or with wide-ranging electrolytes. Both siblings were managed in this manner.

## Conclusions

Since PHA1 was first described, several patients have been reported and our understanding of the disease has continued to expand. This case report offers additional knowledge of a previously unreported novel mutation in the SCNN1B gene found in two Hispanic siblings with PHA1. Further, this case report highlights the significance of genetic counseling and underlines the difficulty of treatment, and offers additional support for the management of this patient population.
